# Constructive Appraisal of “Real-World Study Assessing Sensitivity and Clinical Utility of a Host-Protein Test in Adult Emergency Department Patients With Blood Culture Ordered”

**DOI:** 10.1016/j.acepjo.2025.100319

**Published:** 2026-01-13

**Authors:** Parth Aphale, Himanshu Shekhar, Shashank Dokania

**Affiliations:** Dr. D.Y. Patil Vidyapeeth (Deemed to be University), Pimpri, Pune, Maharashtra, India

To the Editor:

We read with interest the recent study by LoVerde et al^1^ evaluating the diagnostic performance and clinical utility of the MeMed BV host-protein test in adult emergency department patients with suspected infection. The authors demonstrated impressive sensitivity (96.4%) and negative predictive value (98.6%) for distinguishing bacterial from viral infections and reported improved antibiotic alignment over time. Although this real-world investigation contributes meaningfully to diagnostic stewardship, several methodological and interpretive considerations warrant further clarification.

First, the reliance on blood cultures as the reference standard, although practical, is limited by low sensitivity and potential contamination, which may overestimate measured test performance. Incorporating a composite clinical standard including adjudication, imaging, and biomarkers would provide a more reliable benchmark.^2^

Second, the observed improvement in antibiotic alignment may be influenced by unmeasured confounders such as ongoing stewardship initiatives, seasonal infection patterns, or policy changes. Adjustment using multivariable or interrupted time-series methods would clarify the independent effect of the test.

In conclusion, LoVerde et al^1^ provide encouraging real-world evidence supporting the clinical utility of the MeMed BV assay in the emergency department. Addressing limitations related to reference standard selection and potential confounders in prescribing analysis will further strengthen the understanding of its diagnostic and stewardship impact.

## Funding and Support

By *JACEP Open policy*, all authors are required to disclose any and all commercial, financial, and other relationships in any way related to the subject of this article as per ICMJE conflict of interest guidelines (see www.icmje.org). The authors have stated that no such relationships exist.

## Declaration Of Generative AI and AI-Assisted Technologies In The Writing Process

The authors declare that AI-based language assistance was used solely for grammar refinement. All intellectual content, interpretation, and conclusions are the sole responsibility of the authors.

## Conflict of Interest

All authors have affirmed they have no conflicts of interest to declare.
